# Integrated microscale immiscible phase extraction and isothermal amplification for colorimetric detection of *Neisseria gonorrhoeae*

**DOI:** 10.1007/s00216-023-04734-3

**Published:** 2023-05-18

**Authors:** Pablo Rodriguez-Mateos, Bongkot Ngamsom, Daglus Ameyo, Patrick Wakaba, Clement Shiluli, Alexander Iles, Jesse Gitaka, Nicole Pamme

**Affiliations:** 1 Department of Materials and Environmental Chemistry, Stockholm University, Stockholm, Sweden; 2 Centre for Biomedicine, Hull York Medical School, University of Hull, Hull, UK; 3 Directorate of Research and Innovation, Mount Kenya University, Thika, Kenya; 4 School of Natural Sciences, University of Hull, Hull, UK

**Keywords:** Immiscible filtration, DNA extraction, Magnetic particle, NAAT, LAMP, *Neisseria gonorrhoeae*

## Abstract

Gonorrhea is the second most common sexually transmitted infection (STI) with around 87 million cases worldwide estimated in 2016 by the World Health Organization. With over half of the cases being asymptomatic, potential life-threatening complications and increasing numbers of drug-resistant strains, routine monitoring of prevalence and incidence of infections are key preventive measures. Whilst gold standard qPCR tests have excellent accuracy, they are neither affordable nor accessible in low-resource settings. In this study, we developed a lab-on-a-chip platform based on microscale immiscible filtration to extract, concentrate and purify *Neisseria gonorrhoeae* DNA with an integrated detection assay based on colorimetric isothermal amplification. The platform was capable of detecting as low as 500 copies/mL from spiked synthetic urine and showed no cross-reactivity when challenged with DNAs from other common STIs. The credit card–size device allows DNA extraction and purification without power or centrifuges, and the detection reaction only needs a low-tech block heater, providing a straightforward and visual positive/negative result within 1 h. These advantages offer great potential for accurate, affordable and accessible monitoring of gonorrhea infection in resource-poor settings.

**Supplementary Information:**

The online version contains supplementary material available at 10.1007/s00216-023-04734-3.

## Introduction

Gonorrhea is the second most common sexually transmitted infection (STI) with approximately 87 million infections per year globally estimated in 2016 and with the World Health Organization African Region (WHOAR) reporting the highest prevalence and incidence in both men and women [1, 2]. The lifetime direct medical cost attributed to gonorrhea in the USA alone in 2008 was estimated to be $162.1 million [3], with over 600,000 cases being reported in the USA in 2019 (a 92% increase since the historic low in 2009) [4]. Over 55% of gonococcal infections are asymptomatic [5, 6]; however, untreated gonorrhea can lead to complications such as painful urination, urethritis, epididymitis and pelvic inflammatory disease and can result in ectopic pregnancies, infertility and increased risk of HIV [7–9]. The US Center of Disease Control and Prevention (CDC) classified the drug-resistant *Neisseria gonorrhoeae* in the highest category in their 2019 antibiotic resistance report as an urgent threat due to increasing resistance over time from 2000 to 2017, with cephalosporins being the only class of antibiotic recommended for treatment of *N. gonorrhoeae* infections [9, 10]. For these reasons, monitoring the prevalence and incidence through routine screening is a key preventive measure assisting response and treatment choices.

The main diagnostic tests for gonorrhea are summarized and compared in Table 1. Real-time polymerase chain reactions (qPCR) have long been cleared by the Food and Drug Administration (FDA) for the detection of urogenital infections caused by *Neisseria gonorrhoeae* and *Chlamydia trachomatis* [8]. These were recommended as screening or diagnostic tests for patients both with and without symptoms due to their excellent sensitivity and specificity, usually above 95% for both depending on specimen type collected [5, 11]. In contrast with culture methods, nucleic acid amplification tests (NAATs) do not require viable organisms, resulting in easier specimen transport. This has allowed less invasive specimen collection such as first catch urines and self-taken vaginal swabs to detect shed organisms, facilitating disease screening. The sample preparation steps of cell lysis and DNA extraction can also be automated, where the user only introduces the sample in a cartridge format [11, 12]. However, these pieces of equipment are very expensive and oftentimes only specialized technicians can operate them. Additionally, they are mostly accessible to big, centralized laboratories and they are not readily available in low- and middle-income countries, where the services are limited and patients might not be able to pay to access these services [13]. Antigen assays in the format of lateral flow tests can be quick, specific and relatively equipment free, which makes them excellent candidates for community testing purposes. However, their main drawback is low sensitivity, requiring relatively high loads of 10^4^–10^5^ bacteria for the test to become positive. When using urine samples, some tests need a prior centrifugation step to concentrate bacteria, which adds another step and piece of equipment [13]. Loop-mediated isothermal amplification (LAMP) utilizes a single temperature, can achieve faster amplification times than PCR and involves no expensive instrumentation, showing great potential as a NAAT method for routine screening of *N. gonorrhoeae* infections in resource-limited settings.

**Table 1:** Summary of the main tests available for diagnostic of gonorrhea and characteristics. Table adapted with information from [11–13]

Test parameters	Microbial culturing	Antigen detection	NAATs
Affordable	+ +	+ + (US$ 6–7)	+
Sensitive^a^	+ + +	+ + (< 50%)	+ + + (> 97%)
Specific^a^	+ + +	+ + (> 98%)	+ + + (> 99%)
User-friendly	+	+ + (6–7 steps)	+
Rapid and robust	+	+ + (< 60 min)	+ (2–6 h)
Equipment free	No	Yes	No
Other advantages	Drug resistance monitoring	Community testing	Gold standard (qPCR)

^a^Compared to a laboratory-based reference standard assay

LAMP assays for detection of *N. gonorrhoeae* DNA have been developed by different groups, typically targeting the open reading frame (ORF1) of the glutamine synthetase (*glnA*) gene [14, 15], porA pseudogene [16, 17] or penA gene [18, 19]. These assays reported various readout methods such as conventional gel electrophoresis [14], real-time reading of fluorescence signal [16, 17], end point reading of UV fluorescence [18, 19] and real-time turbidity, visual color change with malachite green indicator and visual lateral flow [15]. One of these assays tolerated urea concentrations higher than those present in human urine, showing promise for detecting target nucleic acids from urine samples that had undergone little to no extraction [14]. Other assays focused on designing LAMP primers to detect strain variants that might be resistant and susceptible to treatment with specific antimicrobials [18, 19]. Most assays reported good sensitivities relevant to loads present during infection and good specificities against other bacteria and *Neisseria* strains tested with typical amplification times being around 30–60 min.

In most of these studies, the common bottlenecks were prior cell lysis and DNA extraction steps using kits before amplification reactions. When using urine clinical samples, centrifugation of a few millilitre sample was typically carried out to concentrate and wash the cells from the matrix containing potential inhibitors. These non-integrated sample preparation steps slow down the overall turnaround time and depend on peripheral infrastructure of such centrifuges and extraction kits, which often contain proprietary compositions. Although isothermal amplification assays using fluorescent dyes or target-specific probes with labelled fluorophores may have an increased sensitivity, provide quantitative results and demonstrate potential for multiplexing, they are often run on already available qPCR systems. Although amplification can be performed using simple heating equipment, a fluorescence reader is still required, which can sometimes be expensive and might not always be accessible in resource-poor settings.

A series of approaches using immiscible phases constrained in microscale dimensions together with magnetic microparticle actuation have been developed over the years [20] with names such as immiscible phase filter (IPF) [21], immiscible filtration assisted by surface tension (IFAST) [22] or oil immersed lossless total analysis system (OIL-TAS) [23]. These utilize functionalized paramagnetic particles to capture bioanalytes of interest (i.e. nucleic acids [24–26], proteins [27–29] and whole cells [30–33]) and extract, concentrate and purify them from a matrix sample through a series of immiscible phases. In the context of nucleic acids, in recent years, these platforms have integrated extraction and purification steps with further on-chip amplification and detection assays such as colorimetric (RT)-LAMP to identify DNA from endangered rhino species from dung samples [34] and SARS-CoV-2 RNA from sputum and swab samples [23, 35]. These flexible platforms allow users to streamline and integrate extraction of nucleic acids from complex matrices without a power supply and detection using a simple heating device such as a block heater, showing great potential as point-of-care diagnostic tests for resource-limited settings.

**Fig. 1:**
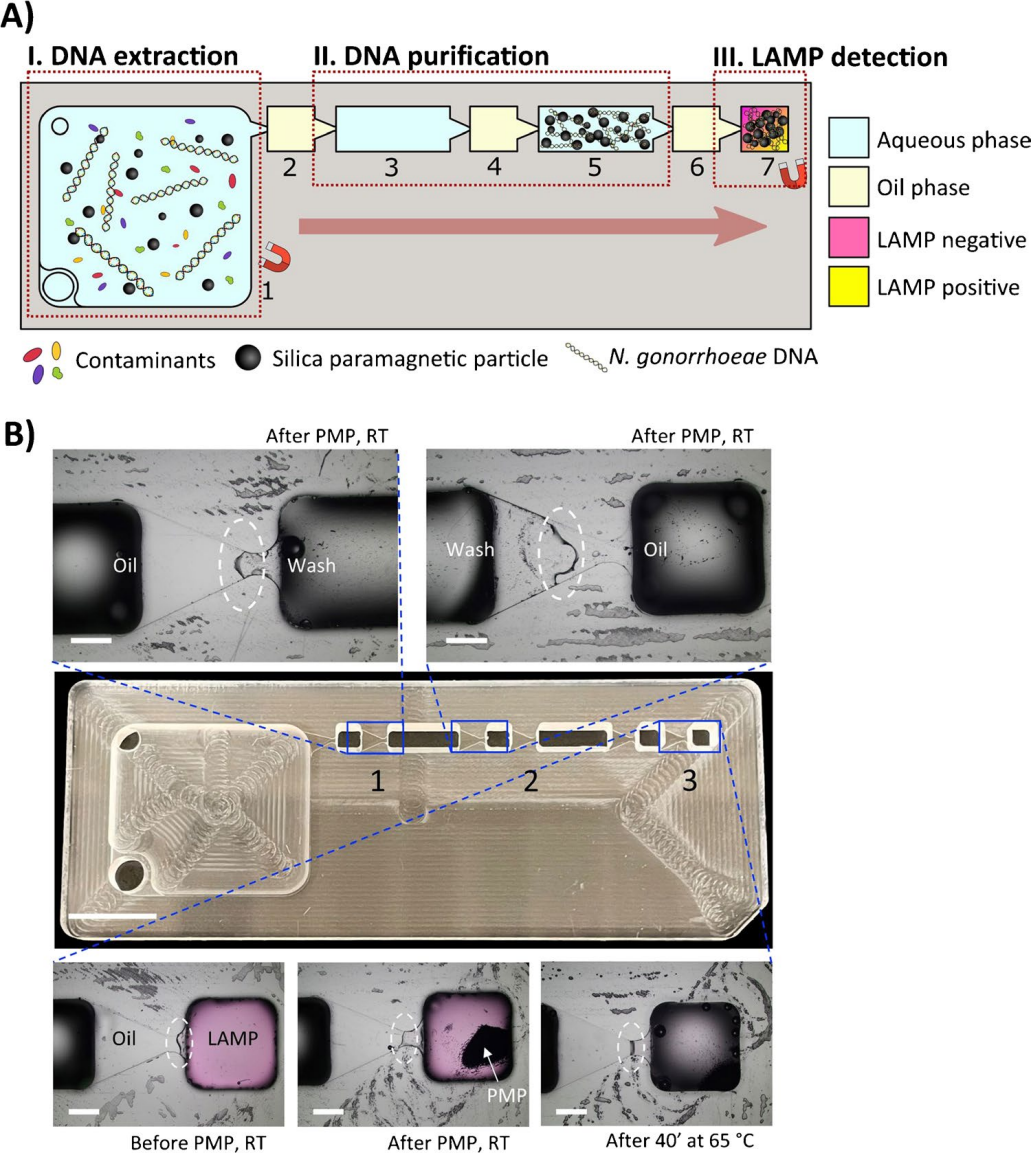
IFAST-LAMP platform for detection of *Neisseria gonorrhoeae* DNA. **A** Schematic workflow of (I) DNA extraction via silica paramagnetic particles (PMP) and GuHCl, (II) concentration and purification through immiscible chambers and (III) detection via colorimetric LAMP assay (color change from pink to yellow for positive amplifications). The device consists of a 1-mL sample chamber (1), three small oil chambers (2, 4, 6) connected by two longer wash chambers (3, 5) and a final LAMP detection chamber (7). **B** Photograph of an 8 × 3 × 0.4 cm polymethyl methacrylate device and microscopic photographs of immiscible barriers inside gates: (1) oil-wash chambers; (2) wash-oil chambers and (3) oil-LAMP chambers before and after passing of PMP at room temperature (RT), and after heating at 65°C for 40 min. Scale bar in PMMA device photograph = 1 cm. Scale bars in microscope gate images = 1 mm

Here, we present a lab-on-a-chip platform based on IFAST and a colorimetric LAMP assay to extract and detect genomic *N. gonorrhoeae* DNA. The approach integrates the consecutive steps of DNA extraction, concentration, purification, amplification and detection with colorimetric readout and naked eye qualitative result interpretation in a single hand-held device (Fig. 1). Our demonstration of the platform’s viability for *N. gonorrhoeae* detection from urine samples may provide an accurate and accessible approach for any sample-to-answer NAATs for point-of-care diagnostics, in particular for resource-poor settings.

## Materials and methods

### Ethical approval

This study was approved by the Mount Kenya University Independent Ethical Review Committee (MKU/IERC/1649) and performed in accordance with relevant guidelines and regulations. Vaginal swabs and urine samples were collected from participants with their written informed consent after the nature and possible consequences of the study had been fully explained to them.

### Reagents

Genomic DNAs from *Neisseria gonorrhoeae* (ATCC 700825DQ), *Chlamydia trachomatis* Serovar D strain UW-3/Cx (ATCC VR-885D), *Trichomonas vaginalis* (ATCC 30001DQ) and *Treponema pallidum* (ATCC BAA-2642SD) and heat-inactivated *N. gonorrhoeae* cells (ATCC 19424-IN) were purchased from LGC standards, UK. WarmStart® colorimetric LAMP 2 × master mix (DNA & RNA) was purchased from New England Biolabs. Primers were supplied from Integrated DNA Technologies (IDT). MagneSil® paramagnetic particles were purchased from Promega. SYBR Safe, mineral oil, Tween 20, DNA decontaminant solution, PCR adhesive film and nuclease-free water were supplied from Thermo Fisher Scientific. Guanidine hydrochloride was purchased from VWR. Sigmatrix synthetic urine diluent was procured from Sigma-Aldrich.

### Device design, fabrication and preparation

The present device has been redesigned from the previous iteration using colorimetric LAMP [35] to allow a quicker purification step by having the chambers aligned in a straight path for easier magnetic manipulation, multiplexing and automation. It has also been simplified in terms of number of steps, complexity and with an overall cost lower than the most recent design combining the dual LAMP and CRISPR assays [36]. Devices were fabricated from polymethyl methacrylate (PMMA) via CNC machine milling (Datron M7). The device featured a sample chamber (1) (*w* = 20 mm, *l* = 23 mm); wash chambers (3, 5) (*w* = 3, *l* = 8.5 mm); oil chambers (2, 4, 6) (*w* = 3 mm, *l* = 3 mm) and a LAMP chamber (*w* = 3 mm, *l* = 3 mm) (Fig. 1A). All chambers had a height of 3.8 mm and were interconnected via gates with the same dimensions as previous iterations (*w* = 3 mm to 0.5 mm, *l* = 3 mm, *h* = 0.2 mm) [35, 36] due to their ability to compartmentalize immiscible liquids side-by-side and provide stable interfaces between aqueous and oil phases at 65°C (Fig. 1B). Devices were cleaned with a DNA decontaminant solution followed by rinsing with deionized water and left to dry at ambient temperature. The bottoms of devices were sealed with PCR adhesive film.

### LAMP assay

The sensitivity of the colorimetric LAMP assay and the primer specificity were first evaluated in tubes. Ten-fold serial dilutions of genomic *N. gonorrhoeae* DNA (from an initial concentration of 5 × 10^5^ copies/µL) were performed in nuclease-free water. Primers targeting the porA pseudogene of *N. gonorrhoeae* reported by Liu et al. [16] were used here (Table S1). A 10 × LAMP primer mix was first prepared following the composition suggested in the LAMP manufacturer guidelines [37]: 16 µM each of forward inner primer (FIP) and backward inner primer (BIP), 2 µM each of forward outer primer (F3) and backward outer primer (B3), and 4 µM each of forward loop primer (LF) and backward loop primer (LB). A typical 20-µL tube-based LAMP reaction contained 2 µL of 10 × LAMP primer mix, 7 µL H_2_O, 10 µL LAMP substrate and 1 µL DNA template at the desired concentration. For no template control (NTC), 1 µL H_2_O was added instead of DNA template. Amplification reactions were carried out at 65°C for 30–60 min (times specified in the “Results and discussion” section) on a pre-warmed block heater (SBH200D, Stuart). After amplification, tubes were left to cool to room temperature for color intensification (pink = negative, yellow = positive) and photographed under normal laboratory lighting conditions with an iPhone 12 mini camera. For confirmation of amplification-dependent color change, LAMP products were electrophoresed in 1% w/v agarose gel stained with SYBR Safe at 80 V for 45 min. Pre- and post-amplification steps were performed in separated spaces and regularly cleaned with DNA decontaminant solution. Gels were imaged in an azure biosystems c600 gel imager. The specificity of the assay was tested by replacing the genomic *N. gonorrhoeae* DNA by equal volumes of genomic *C. trachomatis*,* T. vaginalis* and *T. pallidum* DNAs and compensating with lower volumes of water to obtain a final 20-µL reaction volume.

### Tube-based DNA extraction

DNA extraction using silica paramagnetic particles (PMP) in the presence of guanidine hydrochloride was tested in 1.5-mL tubes. PMP were first washed three times with nuclease-free water and resuspended in the same initial volume with water, typically 100 µL was prepared and sedimented PMP were always resuspended via pipetting before use. As a sample matrix, a total volume of 1 mL of either aqueous 5 M GuHCl containing 0.005% Tween 20 or synthetic urine containing 5 M GuHCl and 0.005% Tween 20 spiked with 1 µL of DNA, heat-inactivated *N. gonorrhoeae* cells or nuclease-free water (for non-template control) and 1.5 µL PMP was prepared. Subsequently, mixing was conducted via tube inversion for 5 min. DNA-bound PMP were collected and kept at the bottom of the tube by the adjacent placement of a neodymium iron boron (NdFeB) magnet assembly. Supernatant was discarded, and the remaining PMP were gently washed with 100 µL nuclease-free water, resuspended in 20 µL LAMP mix (2 µL 10 × LAMP primers, 8 µL H_2_O and 10 µL LAMP substrate) and transferred to a PCR tube for amplification in a block heater as explained earlier for tube-based LAMP assay.

### Integrated on-chip DNA extraction and LAMP detection

For DNA extraction in the IFAST device, a total volume of 1 mL of either aqueous 5 M GuHCl containing 0.005% Tween 20, synthetic or real urine containing 5 M GuHCl and 0.005% Tween 20 spiked with 1 µL NG DNA template (or 1 µL from each *C. trachomatis*, *T. vaginalis* and *T. pallidum* DNAs in case of testing primer specificity) and 1.5 µL PMP was prepared. Subsequently, the 1-mL sample was pipetted into the IFAST sample chamber and the device was manually mixed via circular motion against a flat surface for 5 min. Next, the remaining chambers were filled as follows (Fig. 1A): 30 µL oil in chamber 6, 20 µL LAMP mix (containing 2 µL 10 × LAMP primers, 8 µL H_2_O and 10 µL LAMP substrate) in chamber 7, 30 µL oil in chambers 2 and 4, 50 µL aqueous solution with 0.005% Tween 20 in chambers 3 and 5. Chambers 3 and 5 were overlaid with 30 µL oil and chamber 7 with 10 µL oil. Afterwards, DNA-bound PMP were concentrated and collected from the sample chamber by placing a magnet at the bottom of the device and were purified from the sample matrix through the aqueous/oil barriers until the final chamber 7. Finally, the device was placed on top of a block heater at 65°C for 40–60 min. After amplification, the content was pipetted out from chamber 7 and loaded on a gel electrophoresis as described above. For urine samples tested at Mount Kenya University, LAMP products were run on 1% agarose gels with ethidium bromide at 100 V for 40 min. The complete IFAST-LAMP workflow demonstration can be found in the Supplementary Information section, SI video.

## Results and discussion

### Sensitivity and specificity of tube-based colorimetric LAMP assay

**Fig. 2:**
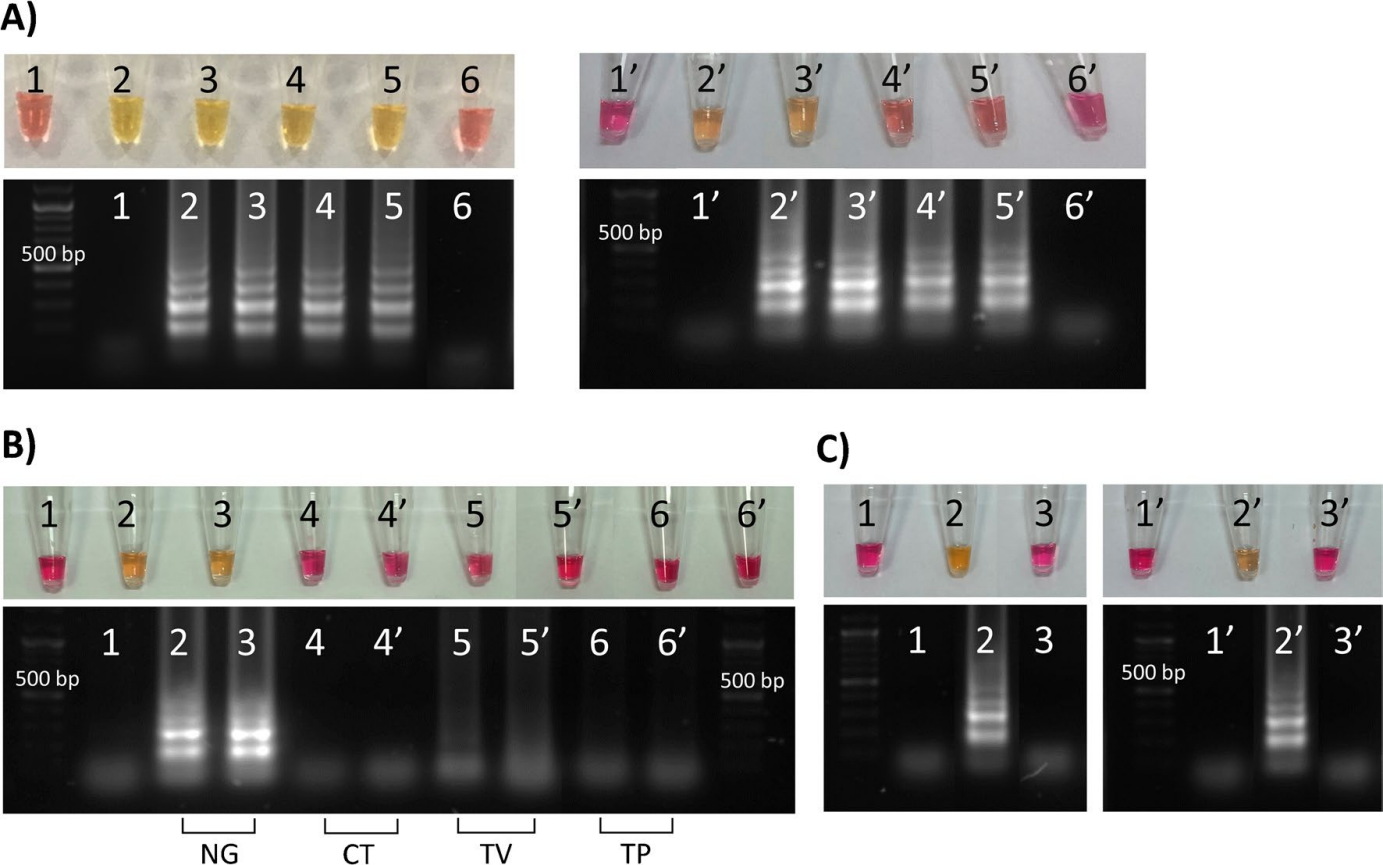
Investigation of tube-based colorimetric LAMP assay for detection of *N. gonorrhoeae* (NG) DNA. **A** Sensitivity of LAMP assay, two replicates of: 1, 1′ = no template control; 2, 2′ = 5 × 10^4^ NG copies; 3, 3′ = 5 × 10^3^ NG copies; 4, 4′ = 500 NG copies; 5, 5′ = 50 NG copies; 6, 6′ = 5 NG copies. Tubes were incubated at 65°C for 35 min. **B** Specificity testing of primers targeting porA pseudogene of NG with single DNAs: 1 = no template control; 2 = 5 × 10^3^ NG copies; 3 = 500 NG copies; 4, 4′ = 1.12 ng *C. trachomatis* (CT) DNA; 5, 5′ = 5 × 10^4^ copies *T. vaginalis* (TV) DNA; 6, 6′ = 4 × 10^4^ copies *T. pallidum* (TP) DNA. Tubes were incubated at 65°C for 30 min (2–3), 40 min (4, 4′) or 50 min (1, 5–6′). **C** Specificity testing of primers targeting porA pseudogene of NG with mixture of DNAs, two replicates of: 1, 1′ = no template control; 2, 2′ = 500 NG copies + 112 pg CT + 5 × 10^3^ TV copies + 4 × 10^3^ TP copies; 3, 3′ = 1.12 ng CT + 5 × 10^4^ TV copies; 4 × 10^4^ TP copies. Tubes were incubated at 65°C for 40 min

The effectiveness of the commercially available colorimetric LAMP assay with primers targeting porA pseudogene for detection of *N. gonorrhoeae* DNA was first evaluated on a series of ten-fold dilutions performed on the initial genomic DNA (5 × 10^5^ copies/µL) in tube-based reactions in duplicate. The assay, based on phenol red color change during amplification-dependent pH drop, was capable of detecting down to 50 copies of the genomic DNA after 35 min (Fig. 2A). This was comparable to the works reported by Edwards et al. [14] (20 copies in 27 min, primers targeting glnA gene, colorimetric LAMP assay), Liu et al. [16] (400 copies in 18 min, primers targeting porA pseudogene, fluorescent LAMP assay) and Eboigbodin et al. [17] (20 copies in 60 min, primers targeting porA pseudogene, fluorescent LAMP assay).

The specificity of the LAMP primers targeting *N. gonorrhoeae porA* was tested in duplicate against genomic DNAs from other common curable STIs, *Chlamydia trachomatis* (CT), *Trichomonas vaginalis* (TV) and *Treponema pallidum* (TP). LAMP assays conducted on these DNAs using corresponding primers showed positive control amplifications (Fig. S1). Primers targeting *N. gonorrhoeae* *porA* showed no cross-reactivity towards other tested DNAs, either when added to each DNA (Fig. 2B) or when added to a mixture of DNAs (Fig. 2C). These results add three new DNAs to the list of 23 bacterial species that do not cross-react with the same LAMP porA primers reported by Liu et al. [16]. They also show the possibility of adapting the herein single-assay device for simultaneous detection of NG, CT, TV and TP at a single amplification temperature and time from one sample.

### Tube-based DNA capture with silica paramagnetic particles

**Fig. 3:**
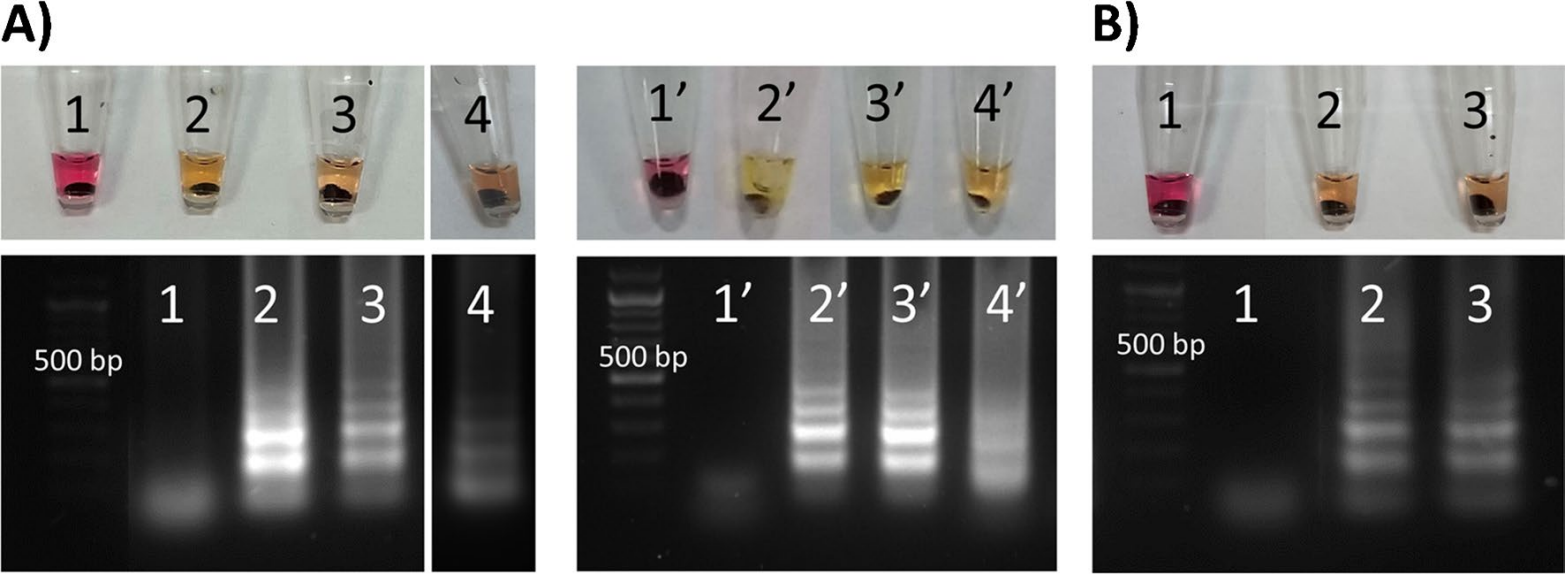
Tube-based extraction of *N. gonorrhoeae* DNA via silica paramagnetic particles followed by tube-based LAMP assay. **A** Free NG genomic DNA in aqueous solution containing 5 M GuHCl, two replicates of: 1, 1′ = no template control; 2, 2′ = 5 × 10^4^ copies/mL; 3, 3′ = 5 × 10^3^ copies/mL; 4, 4′ = 500 copies/mL. Tubes incubated at 65°C for 45 min (1–4, 2′–3′) or 60 min (1′, 4′). **B** Heat-inactivated *N. gonorrhoeae* cells in aqueous solution containing 5 M GuHCl: 1 = no template control; 2 = 2 × 10^4^ copies/mL; 3 = 2 × 10.^3^ copies/mL. Tubes incubated at 65°C for 30 min (*n* = 1)

Silica paramagnetic particles (1–16 µm diameter, Fig. S2A) employed for DNA extraction come as a suspension in storage solution containing GuHCl amongst other proprietary ingredients [38], which can inhibit amplification when directly added to a LAMP reaction. The interference from the liquid suspension can be removed by washing the particles with nuclease-free water prior to adding them to the reaction (Fig. S3). The washed PMP were next evaluated for tube-based extraction of *N. gonorrhoeae* DNA from spiked aqueous solution of GuHCl (1 mL, 5 M, 5 min mixing) in duplicate. Detection of as low as 500 copies/mL was achieved after 45–60 min at 65°C (Fig. 3A). These capture and detection levels were comparable to previous studies using similar paramagnetic particles [35, 36]. Capture and detection of lower copy numbers (≤ 50 copies/mL) were not reliable, and the higher amplification times needed in these cases compared to previous tube-based LAMP assays (Fig. 2A) might be due to the following: (1) suboptimal DNA-PMP capture efficiencies, (2) suboptimal washing of GuHCl matrix leading to partial LAMP inhibition and (3) loss of DNA due to repeat washing of PMP-bound DNA. Tube-based capture of DNA from heat-inactivated *N. gonorrhoeae* cells suspended in 5 M GuHCl was also tested, followed by tube-based LAMP assays. Detection of 2 × 10^3^ copies/mL was achieved after 30-min amplification (Fig. 3B, n = 1). These results confirm the advantage of using PMP to capture and concentrate DNA from samples with low concentrations, which would otherwise have not been possible to detect. Pipetting 1–3 µL of a sample containing 5 × 10^3^ copies/mL into a final 20-µL LAMP reaction would result in approximately 5–15 copies per reaction, lower than or around the sensitivity of the assay (Fig. 2A).

### Integrated on-chip DNA extraction, purification and detection

**Fig. 4:**
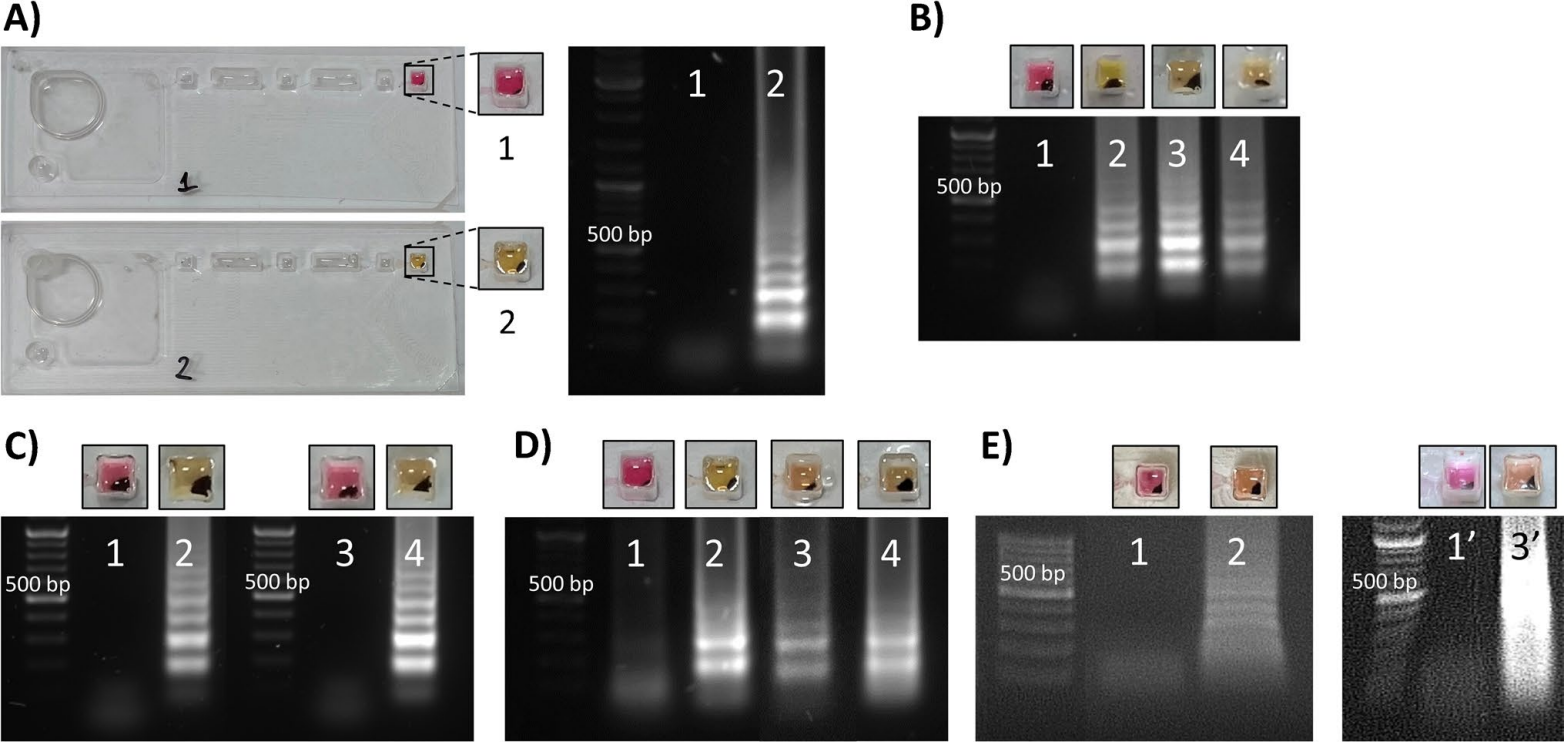
On-chip integrated steps of extraction and colorimetric LAMP detection of *N. gonorrhoeae* (NG) DNA. **A** Proof-of-concept IFAST-LAMP devices: 1 = remaining pink with no amplification for a no template control, 2 = turning yellow and showing amplification for 5 × 10^4^ copies/mL. **B** Aqueous 5 M GuHCl with 0.005% Tween 20 matrix spiked with NG DNA: 1 = no template control; 2 = 5 × 10^4^ copies/mL; 3 = 5 × 10^3^ copies/mL; 4 = 500 copies/mL. Devices incubated at 65°C for 40 min (*n* = 3). **C** Aqueous 5 M GuHCl with 0.005% Tween 20 matrix spiked with mixture of DNAs: 1 = 1.12 ng CT DNA + 5 × 10^5^ TV copies + 4 × 10^5^ TP copies; 2 = 5 × 10^5^ NG copies + 1.12 ng CT DNA + 5 × 10^5^ TV copies + 4 × 10^5^ TP copies; 3 = 0.112 ng CT DNA, 5 × 10^4^ TV copies, 4 × 10^4^ TP copies; 4 = 5 × 10^4^ NG copies + 0.112 ng CT DNA, 5 × 10^4^ TV copies, 4 × 10^4^ TP copies. Devices incubated at 65°C for 40 min (*n* = 1). **D** Sigmatrix synthetic urine containing 5 M GuHCl and 0.005% Tween 20 spiked with NG DNA: 1 = no template control; 2 = 5 × 10^4^ copies/mL; 3 = 5 × 10^3^ copies/mL; 4 = 500 copies/mL. Devices incubated at 65°C for 40 min (*n* = 1). **E** Human urine containing 5 M GuHCl and 0.005% Tween 20 spiked with NG DNA: 1, 1′ = no template control; 2 = 5 × 10^4^ copies/mL; 3′ = 5 × 10^3^ copies/mL. Devices incubated at 65°C for 40 min (1, 2) or 60 min (1′, 3′), *n* = 1

The integrated steps of extraction, purification and detection of *N. gonorrhoeae* DNA were next translated into on-chip assays using the IFAST device. The platform allowed DNA extraction from both aqueous 5 M GuHCl (Fig. 4A, B; *n* = 3) and synthetic urine (Fig. 4D, n = 1), being able to extract and detect 500 copies/mL after 40-min amplification. Recent studies reported mean *N. gonorrhoeae* loads in urine and vaginal swabs to be around 2 × 10^4^ CFU/mL [6]. Other investigations found bacterial loads of 3.7 × 10^6^ and 2 × 10^5^ copies per swab in symptomatic and asymptomatic male urethral infections, respectively [39], and mean bacterial loads in male urine with symptomatic infections of 3.9 × 10^4^ copies/mL [40].

The system was challenged with mixtures of DNAs loaded in the first sample chamber. Specificity to *N. gonorrhoeae* was retained, as cross-reaction to other STI DNAs did not occur (Fig. 4C, n = 1). When testing urine from a healthy participant spiked with *N. gonorrhoeae* DNA, detection of 5 × 10^4^ copies/mL was achieved under 40 min, and 5 × 10^3^ copies/mL in 60 min (Fig. 4E, n = 1). Whilst these bacterial loads are still at a relevant infection level in patients’ urine samples, further investigation and optimization would be beneficial.

Edward et al. showed that LAMP was able to withstand higher concentrations of urea than those found in human urine [14], but most studies still carried out DNA extraction from urine first before LAMP or other amplification reactions. The particular case of the pH-dependent colorimetric LAMP used herein offers great advantages for result visualization and interpretation via the naked eye. The assay works such that protons generated during the exponential amplification reaction acidify the media and a low buffer composition in the master mix containing phenol red allows color change indication [37]. This particular version of the assay; however, could be affected by the wide range of pH in human urine, normal values spanning from pH 4.5 to 7.8 [41], and thus it is essential to extract the DNA from the urine matrix for reliable performance. LAMP assays with other colorimetric readouts have been recently reported and could potentially be incorporated in the current platform [42]. The IFAST-LAMP presented herein allows for integrated steps of DNA capture, concentration and purification from aqueous, synthetic and real urine matrices and simultaneous amplification and detection via colorimetric LAMP assay under 1 h. This flexible platform could additionally incorporate other primers to target antimicrobial resistant or susceptible *N. gonorrhoeae* strains [18, 19].

The next challenges to be investigated include an extensive clinical validation with patient samples and comparison against a gold standard qPCR method. Pre-storage of reagents, either by freeze-drying [36], or by sealing the device to facilitate deployment in a more ready-to-use format shall be the next steps to follow.

## Conclusions

We report a simple and integrated platform based on microscale immiscible filtration and isothermal amplification for colorimetric detection of *N. gonorrhoeae* DNA. This system allows DNA capture from synthetic urine matrices using GuHCl and silica paramagnetic particles, concentration and washing through immiscible aqueous/oil interfaces, and amplification and specific detection of down to 500 copies/mL of target DNA in a single step through an on-chip colorimetric LAMP assay. The under 1 h overall turnaround time, the straightforward nature of the workflow, the low complexity in instrumentation, and easy result interpretation via naked eye readout make this platform a great candidate for monitoring of gonorrhea infections in resource poor-settings.

## Supplementary Information

Below is the link to the electronic supplementary material.

Supplementary file1 (DOCX 1269 KB)

Supplementary file2 (MP4 41873 KB)

## Data Availability

The datasets generated during and/or analyzed during the current study are available from the corresponding authors on reasonable request.
