# Supplementary material for: Integrated microscale immiscible phase extraction and isothermal amplification for colorimetric detection of Neisseria gonorrhoeae
Source: Anal Bioanal Chem. 2023 May 18;415(21):5129–37. doi: 10.1007/s00216-023-04734-3 (PMC10191819; doi:10.1007/s00216-023-04734-3)
Supplement: Supplementary file 1 — Supplementary file1 (DOCX 1269 KB) [file 216_2023_4734_MOESM1_ESM.docx]

## Supplementary information (SI)

**Table S1**. Primer names and sequences used for LAMP.

| Organism (target) | Primer | 5’ - 3’ sequence |
| --- | --- | --- |
| *Neisseria gonorrhoeae* (*porA* pseudogene) [1] | F3 | CCATTGATCCTTGGGACAG |
|  | B3 | CAGACCGGCATAATACACAT |
|  | FIP | GGGAATCGTAACGCACGGAAATAATGTGGCTTCGCAATTG |
|  | BIP | AGCGGCAGCATTCAATTTGTTCCTGATTACTTTCCAGCGTGA |
|  | LF | ATACCGTCGTGGCGTTTG |
|  | LB | CGCCTATACGCCTGCTAC |
|  |  |  |
| *Chlamydia trachomatis* serovar D (CDS2) [2] | F3 | AATATCATCTTTGCGGTTGC |
|  | B3 | TCTACAAGAGTACATCGGTCA |
|  | FIP | TCGAGCAACCGCTGTGACGACCTTCATTATGTCGGAGTC |
|  | BIP | GCAGCTTGTAGTCCTGCTTGAGTCTTCGTAACTCGCTCC |
|  | LF | TACAAACGCCTAGGGTGC |
|  | LB | CGGGCGATTTGCCTTAAC |
|  |  |  |
| *Trichomonas vaginalis* (repeated DNA target) [3] | F3 | ACTATGGCACGAGACACA |
|  | B3 | TTGAAGTGGACACAATCGTT |
|  | FIP | CGAAGTGCTCGAATGCGATTGCATTGACCACACGGACAA |
|  | BIP | GGTGCAAGGCAGAGGTCATTATTGCCAATCCAAGGACG |
|  | LF | GCTGCTTGACCATCCGAA |
|  | LB | GCCACTCTACGAGCAGTAC |
|  |  |  |
| *Treponema pallidum* (bpm gene) [4] | F3 | ACGCCTCCATCGTCAGAC |
|  | B3 | CCGAAGGGTTCAGGTCCT |
|  | FIP | TGCACAGGCGGGTTACTCTGGTGGCAGTAACCGCAGTC |
|  | BIP | AATGTCAGCCGTGGCTTTGACAGCGAAAGCGCAAGAGTTTG |


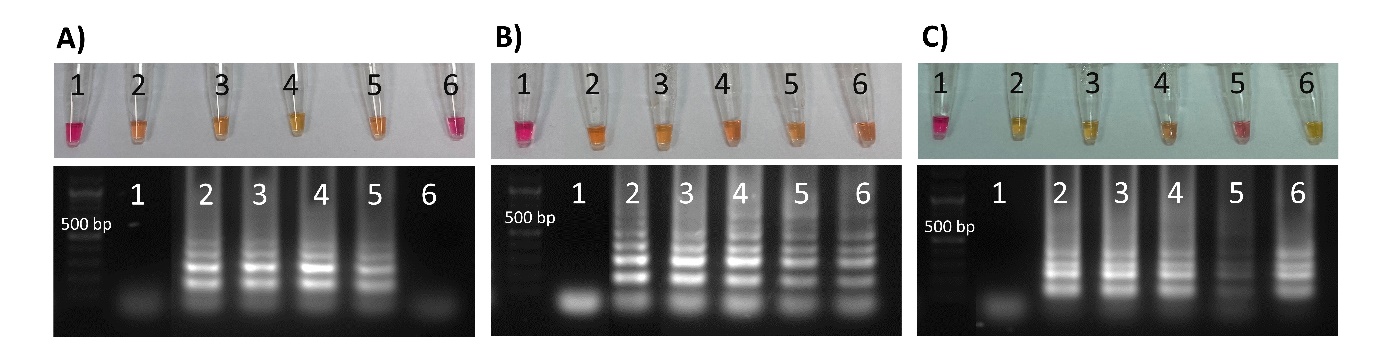


**Fig S1.** Tube-based colorimetric LAMP assay for *C. trachomatis*, *T. vaginalis* and *T. pallidum* DNAs using their respective targeting primers: **A)** Genomic DNA from *C. trachomatis*: 1 = no template control; 2 = 1.12 ng; 3 = 112 pg; 4 = 11.2 pg; 5 = 1.12 pg; 6 = 0.112 pg. Tubes incubated at 65 °C for 35 min (2-5) or 50 min (1, 6). **B)** Genomic DNA from *T. vaginalis*: 1 = no template control; 2 = 9.3 × 10^4^ copies; 3 = 9.3 × 10^3^ copies; 4 = 930 copies; 5 = 93 copies; 6 = 9.3 copies. Tubes incubated at 65 °C for 40 min (2-4) or 45 min (1, 5-6). **C)** Genomic DNA from *T. pallidum*: 1 = no template control; 2 = 7.6 × 10^4^ copies; 3 = 7.6 × 10^3^ copies; 4 = 760 copies; 5 = 76 copies; 6 = 7.6 copies. Tubes incubated at 65 °C for 45 min (2-3) or 50 min (1, 4-6).


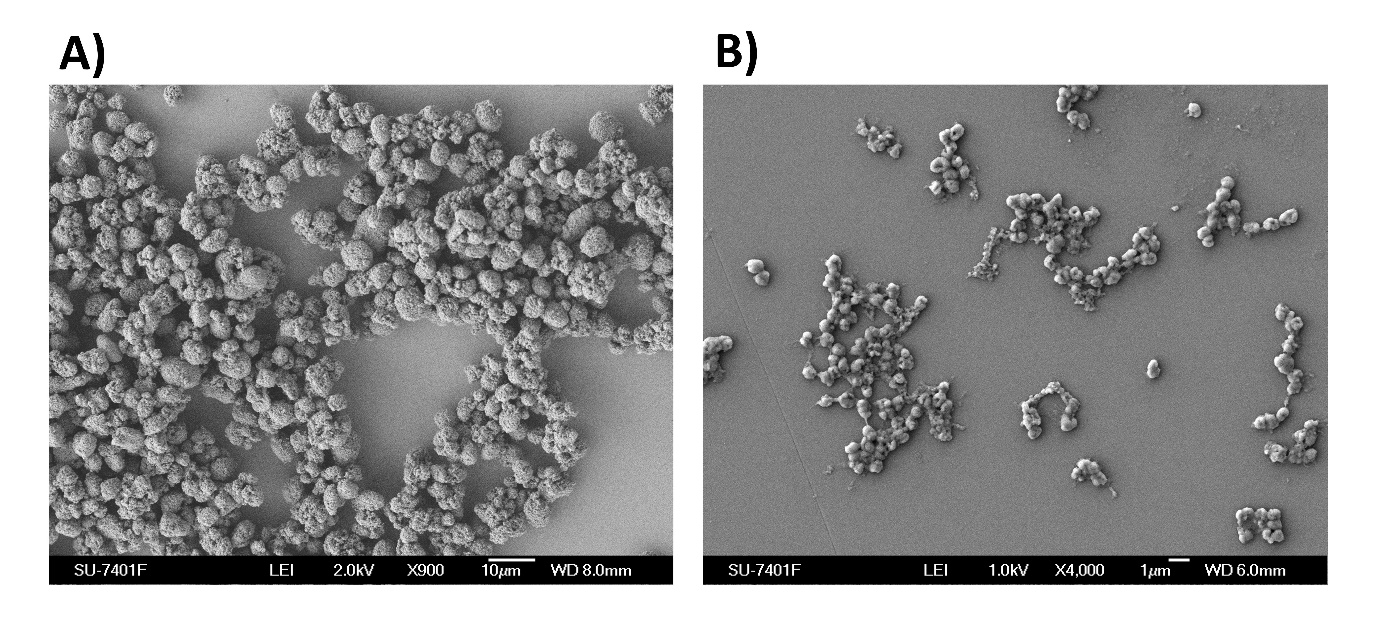


**Fig S2.** Scanning electron microscopy (SEM) images of: **A)** silica paramagnetic particles (PMP) and B) heat inactivated *N. gonorrhoeae* cells. PMP were washed in water and a 10 µL droplet was dried on a silica wafer and visualized directly on an SEM. **B)** *N. gonorrhoeae* cells at a concentration of 10^7^ cells/mL were fixed in 4% paraformaldehyde for 20 min and dehydrated in ethanol (50%, 70%, 90%, 95% and 100% 5 min each). A 10 µL droplet was air dried on a silica wafer and sputter coated with 10 µm gold layer.


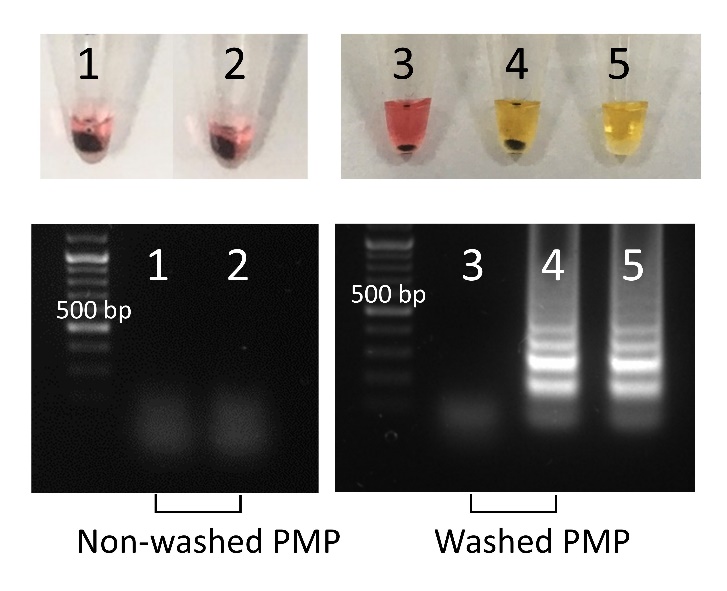


**Fig S3.** Effect of silica paramagnetic particles (PMP) on LAMP reactions. 1 = unwashed PMP with no template control; 2 = unwashed PMP with 4 × 10^3^ NG copies; 3 = washed PMP with no template control; 4 = washed PMP with 4 × 10^3^ NG copies; 5 = 4 × 10^3^ NG copies (positive control without PMP addition). LAMP assays performed at 65 °C for 30 min (3, 4, 5). Non-washed PMP = PMP directly added to the LAMP reaction from the original suspension; washed PMP = PMP washed with nuclease-free water before being added to the LAMP reaction.

**
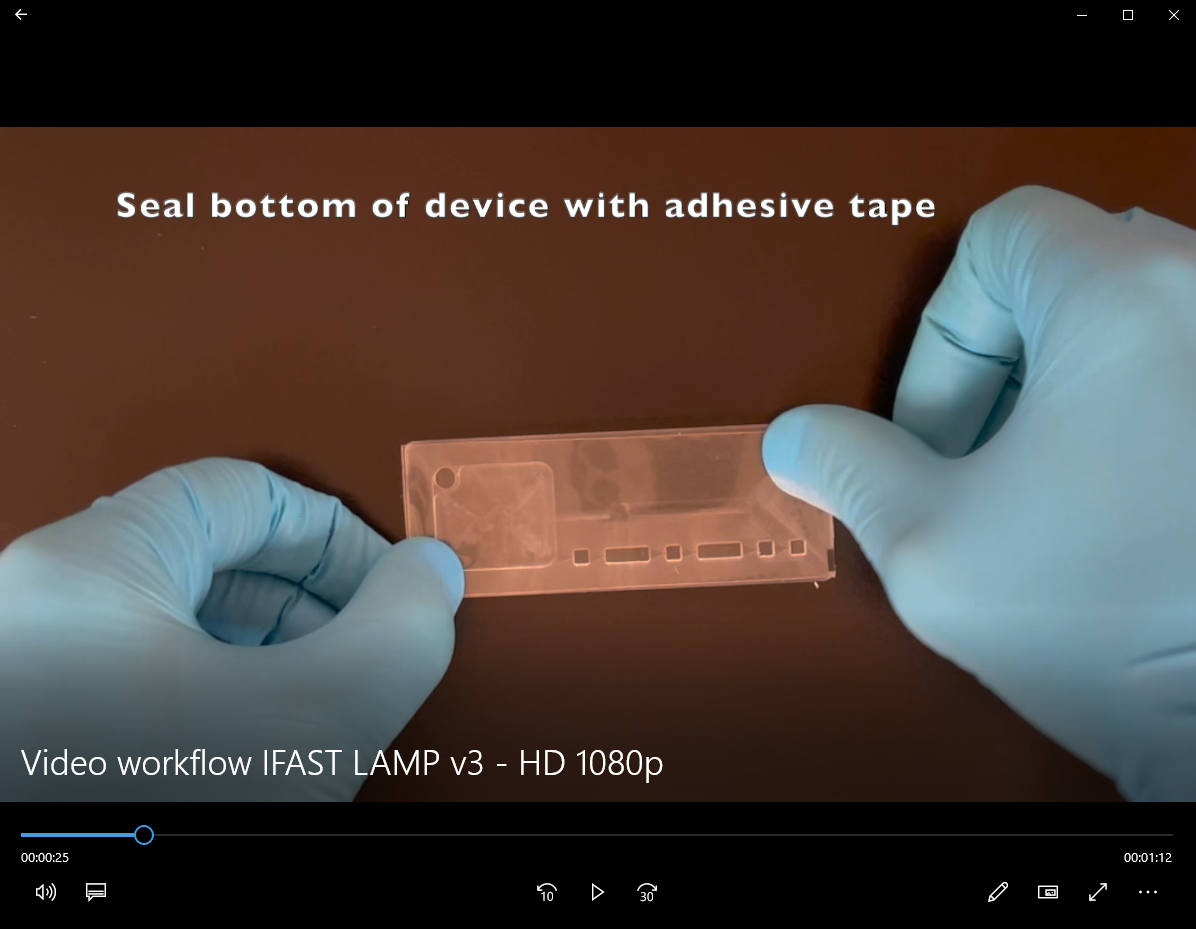
**

**SI video**: Demonstration of the IFAST-LAMP platform workflow for detection of *N. gonorrhoeae* DNA. Video is played at x2 speed. Steps of DNA capture, pipetting and amplification incubation were cut short.

## References

1. Liu ML, Xia Y, Wu XZ, Huang JQ, Guo XG (2017) Loop-mediated isothermal amplification of *Neisseria gonorrhoeae* porA pseudogene: a rapid and reliable method to detect gonorrhea. AMB Express 7 (1):48. doi:10.1186/s13568-017-0349-6

2. Jevtusevskaja J, Uusna J, Andresen L, Krolov K, Laanpere M, Grellier T, Tulp I, Langel U (2016) Combination with antimicrobial peptide lyses improves loop-mediated isothermal amplification based method for Chlamydia trachomatis detection directly in urine sample. BMC Infect Dis 16:329. doi:10.1186/s12879-016-1674-0

3. Reyes JCB, Solon JAA, Rivera WL (2014) Development of a loop-mediated isothermal amplification assay for detection of Trichomonas vaginalis. Diagnostic Microbiology and Infectious Disease 79 (3):337-341. doi:10.1016/j.diagmicrobio.2014.03.016

4. Xiao Y, Xie Y, Xu M, Liu S, Jiang C, Zhao F, Zeng T, Liu Z, Yu J, Wu Y (2017) Development and Evaluation of a Loop-Mediated Isothermal Amplification Assay for the Detection of Treponema pallidum DNA in the Peripheral Blood of Secondary Syphilis Patients. The American Journal of Tropical Medicine and Hygiene 97 (6):1673-1678. doi:10.4269/ajtmh.17-0051
